# Assessment and spatial partitioning of ecosystem services importance in Giant Panda National Park: To provide targeted ecological protection

**DOI:** 10.1371/journal.pone.0278877

**Published:** 2022-12-09

**Authors:** Zhigang Li, Jiaxing Zhu

**Affiliations:** 1 School of Management Science, Chengdu University of Technology, Chengdu, China; 2 Protection Policy Research Center for Key Ecological Functional Areas in the Upper Reaches of the Yangtze River, China; 3 College of Earth Sciences, Chengdu Univ. of Technology, Chengdu, PR China; Feroze Gandhi Degree College, INDIA

## Abstract

Giant Panda National Park is crucial for China’s ecological security strategic pattern known as "two screens and three belts." The importance assessment and classification of ecosystem services in giant panda national parks has an important guiding role in the protection of giant panda national park ecosystems. In this study, we examined four indicators of habitat quality: carbon storage, water conservation, and soil and water conservation. Combined with data analysis were used to evaluate and classify the importance of ecosystem services in the study area. The results showed that: (1) the overall habitat quality index in the study area was relatively high, and the index was generally greater than 0.5. The total carbon storage was 60.5 × 106 t, and the highest carbon storage in the region was 16.9533 t. The area with the highest water conservation reached 715.275 mm. The total soil conservation was 2555.7 × 107 t. (2) From the perspective of spatial characteristics, the habitat quality in the study area presented a spatial distribution pattern of high–low from west to east. The carbon storage presented a spatial distribution pattern of high–low from east to west. The soil conservation presented a spatial pattern of decreasing from west to east, and the water conservation increased from west to east. (3) We divided the research into four levels of importance: The area of general importance in the study site accounted for 1017.58 km^2^ and was distributed in the northwest of the study site. The moderately important areas were distributed in the east of the study site, with an area of 1142.40 km^2^. The highly important areas were distributed in the west of the study site, totaling 2647.84 km^2^. Extremely important areas were distributed in the middle, with an area of 1451.32 km^2^. (4) The grid cell scale of the study area was used as the dataset to determine the weighting. This makes the weighting more objective and ensures that the spatial distribution of areas with different degrees of importance will be more accurate.

## Introduction

The state establishes national parks to achieve scientific protection and rational use of natural resources the main objective is to protect large natural ecosystems with national representation [1–3]. Among them, the Qinling area of Giant Panda National Park is a priority site for biodiversity conservation in China. This is an important area regarding ecological functions, an ecological safety barrier, and a carbon storage and water conservation entity in China [4]. At present, the research on ecosystem services of the Giant Panda National Park is mainly divided into the research on the protection of giant panda species habitat and the research on the spatial optimization of the reserve [5,6]. Habitat protection research aims at species protection, uses species distribution and environmental data, and uses specific algorithms to evaluate the spatial distribution of species [7,8]. The research on spatial optimization of protected areas identifies optimization areas by analyzing specific indicators [9,10]. The distribution of giant pandas often depends on the ecological system of their habitats. However, from the perspective of the overall protection of the Giant Panda National Park, it is difficult to achieve the protection objectives within the entire Giant Panda National Park without comprehensive zoning of the reserve. Based on this, it is necessary to research the importance of ecosystem services in Giant Panda National Park. The research results can be easily used in the decision-making of the ecological division hierarchical management and protection policy of Giant Panda National Park.

Ecosystem service importance assessment and classifying can reveal the quantity and spatial variability of ecosystem services, Identify important areas of ecosystem services in the region, which can reflect the stability and health status of ecosystems from different aspects, which is helpful for hierarchical management and protection [11]. Since the 1990s, ecological zoning has been widely developed and applied in regions, countries, and the world [12]. Zoning system management and environmental protection based on eco-regions have become a hot research topic for environmental managers and policymakers worldwide. For a long time, people have been accustomed to managing and evaluating regional ecological environments based on political-administrative unit and geographical unit division [13]. This single-element local ecological management model has been difficult to effectively and systematically solve ecological and environmental problems. More and more researchers and government agencies recognize the importance of studying and managing natural resources and the environment from the perspective of the whole ecosystem. The regional division based on the ecosystem has become the core of ecosystem management [14]. In existing studies, characterizing the importance of ecosystem services based on a single ecosystem service or a combination of multiple ecosystem services has become the mainstream research paradigm [15–18], a single ecosystem service cannot reflect the ecological status of the entire region, multiple ecosystem services are often more in line with the actual situation of the region, and the research results are more valuable [19,20]. In this study, we based on the actual situation of the region, selected four ecosystem services: habitat quality, soil conservation, carbon storage and water conservation to study. Therefore, by assessing and classifying the importance of regional ecosystem services, it can provide references and suggestions for the follow-up research on my country’s national park eco-regions and the improvement of my country’s ecological function zoning.

In assessing and classifying the importance of ecosystem services, most researchers use the model evaluation method and quantitative index evaluation method. The accuracy of the model evaluation method is relatively high. But the demand for data is large, and the model establishment is rather complex. The quantitative index evaluation method has relatively few parameters, and the operation is relatively simple. However, it cannot objectively express the service function of the ecosystem, research has high uncertainty and one-sidedness [21–26]. When classifying the importance of regions, most scholars artificially classify the results according to the numerical size of the results. which makes the final result lack accuracy [27,28]. In order to improve the accuracy of the results, this study based on the existing research, reduce the size of the study area and use a high-resolution grid cell for the study. Grid cells are readily available, and their matrix format facilitates computation, raster processing, and visualization of results through spatial data management, grid generation, data interpolation, and boundary fitting. The grid-based geographic information system (GIS) can facilitate the assessment of the importance of ecosystem services to reduce the deviation between the results and the actual values [29–31]. In recent years, scholars have carried out several studies on assessment and classifying the importance of ecosystem services based on the InVEST model [32–34]. The InVEST model has been widely used internationally due to the high degree of visualization of the evaluation results. However, it is difficult to obtain relevant parameters, and the simulation accuracy is low, which may lead to more significant uncertainty in the evaluation results [35]. Therefore, we introduce an objective entropy weight method, extract grid data, and construct a framework for assessing and classifying the importance of ecosystem services. It makes the evaluation results more accurate and reduces the error of the evaluation results to a certain extent. At present, most scholars use the analytic hierarchy process and expert knowledge method to determine the weight of ecosystem services [36]. Compared with these subjective assignment methods, the entropy weight assignment method is more accurate and objective and can better explain the results [37].

Therefore, this study divides ecologically important areas based on the evaluation of the ecosystem services quality of Giant Panda National Park. According to different important areas, develop corresponding protection and management measures to improve the efficiency of ecological protection and management.

## Materials and methods

### Study area

**Fig 1:**
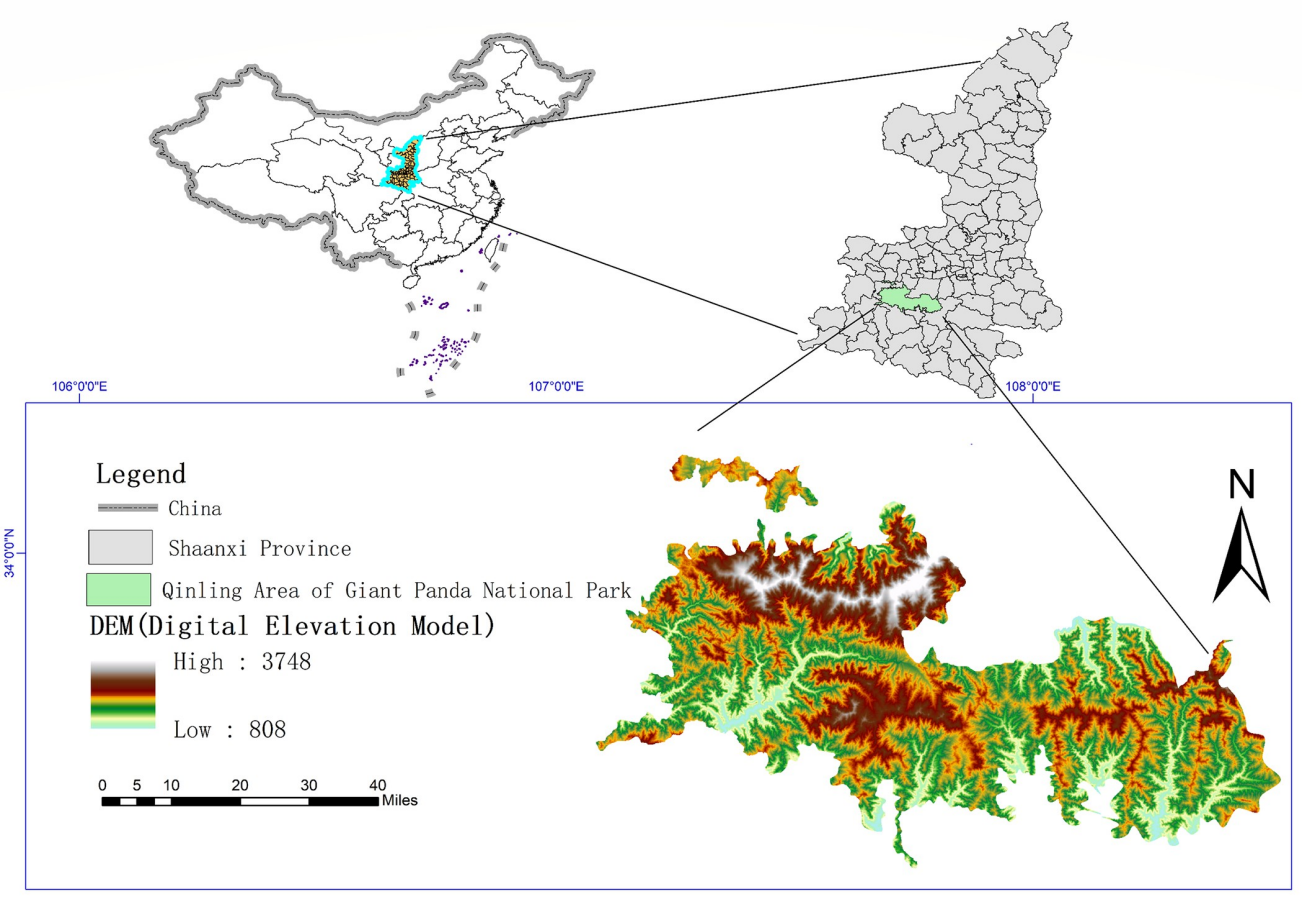
Geographical location of the study area.

Giant Panda National Park is located in western China, consisting of the Minshan District of Sichuan Province, Qionglai Mountain-Daxiangling District of Sichuan Province, Qinling District of Shaanxi Province, and Baishuijiang District of Gansu Province, with a planned area of 27134 square kilometers [38,39]. Qinling District covers four cities and eight counties, including Baoji, Hanzhong, Xi ’an, and Ankang. It is the region with the highest density of wild Giant Pandas in China. It belongs to the monsoon climate zone of the transition from the continental north subtropics to the warm temperate zone, and the forest coverage rate is 72.07%. There are five river systems in the region: Jialing River, Minjiang River, Tuojiang River, Han River, and Weihe River in the Yellow River. The pilot area is 224 square kilometers, and the wetland rate is 0.83%. The Qinling Area of Shaanxi province covers an area of 438600 hectares, covering more than 70 percent of the Qinling Giant Panda habitat. The core protected area is 315100 hectares, and the general control area is 123500 hectares. There are 298 Giant Pandas distributed in 19 towns and villages of xi ’an, Baoji, Hanzhong, and Ankang, including 12 nature reserves, two forest parks, two water conservancy scenic spots, three provincial forestry bureau, and 16 forest farms. The study area has large topographic fluctuations, with the highest elevation of 3748 meters and the lowest elevation of 808 meters, and a relative elevation difference of 2940 meters (Fig 1) [40–43].

### Data sources

The data used in this paper mainly include DEM (Digital Elevation Model) data, land use cover data, meteorological data, soil data, hydrological data, road data, and potential evapotranspiration data. In order to ensure the timeliness and integrity of the data, this paper uniformly selects the data in 2018. DEM data came from the geospatial data cloud platform of the Computer Network Information Center, Chinese Academy of Sciences, with a spatial resolution of 90 meters. The downloaded DEM data were splicing, projection conversion, clipping, and other operations. The land use cover data were obtained from the Data Center for Resources and Environmental Sciences, Chinese Academy of Sciences. The downloaded land use cover data included six first-level and 25 second-level types. Meteorological data sources and China Meteorological Data Network, the data of 49 meteorological stations in Shaanxi province in 2018 were downloaded, and the Spatial distribution map of rainfall was generated by Kriging interpolation using ArcMap, and then the research area was clipped. Hydrological data were extracted from DEM data using hydrological analysis tools of ArcMap. Road data comes from vector data of BIGEMAP. The soil data came from the Cold and Arid Regions. Scientific Data Center: China soil data set based on the world Soil Database (HWSD); Potential evapotranspiration (ETO) data were obtained from the Global Drought and PET databases (Table 1).

**Table 1:** Data source table.

Data name	Data accuracy	Data source / time
DEM data	30*30m	Geospatial data cloud platform of computer network information center of Chinese Academy of Sciences/2018
Land use data of Shaanxi Province	30*30m	Data center of resources and environment science, Chinese Academy of Sciences/2018
Meteorological data of Shaanxi Province	Site data	China Meteorological Data Network/2018
Soil data	1km	Scientific data center for cold and dry regions: Chinese soil data set based on world soil database (HWSD) /2018
Potential evapotranspiration data	1km	Global drought and PET database/2018
Road vector data	-	BIGEMAP/2018
Basic geographic data	-	China National Geographic Information Center

### Framework or assessing the importance of ecosystem services in national parks

**Fig 2:**
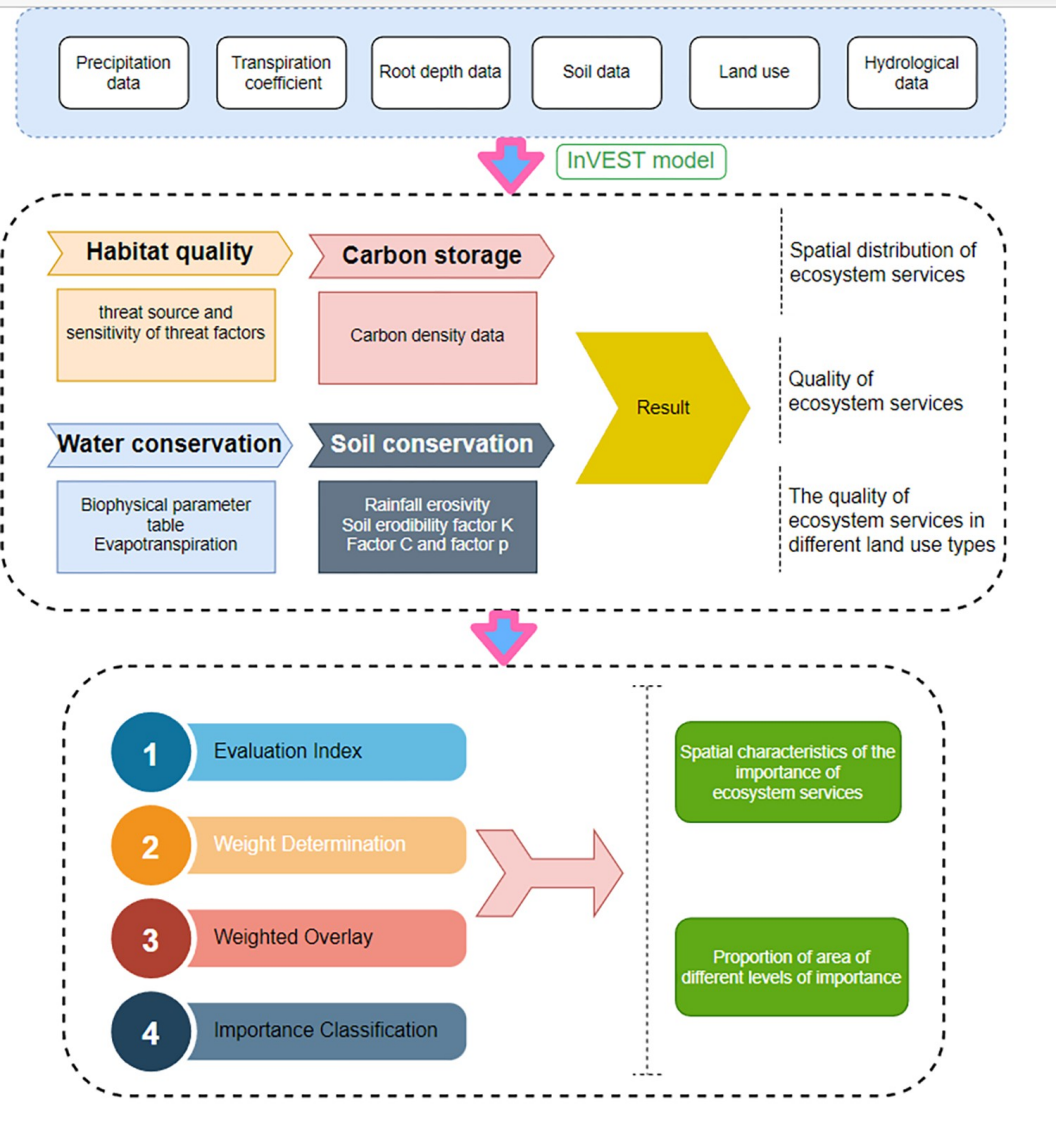
Framework diagram of ecosystem service function importance assessment.

The InVEST model was jointly developed by Stanford University, the Nature Conservancy, and the World Wildlife Fund. The model includes modules of water yield, soil conservation, water purification, habitat quality, carbon storage, and other ecosystem attributes [44–46]. It is a comprehensive assessment model that can quantify various ecosystem services. Compared with the previous evaluation methods for ecosystem services, the most significant advantage of this model is the visual expression of evaluation results that solves the problems associated with previous evaluation models of ecosystem service evaluation being too abstract and unintuitive. Moreover, the visualized evaluation results provide an intuitive spatial distribution; the model also uses ArcGIS to extract attribute values as a dataset for determining weights by the entropy weight method. By extracting raster attribute values, all attribute values are used as a dataset to determine the weights. To make the evaluation results closer to the actual situation of the study area, we chose the entropy weight method to determine the weights and combined these using the weighted sum formula to classify the importance. The framework of ecosystem service functional importance assessment is shown in Fig 2.

### InVEST model

(1) Habitat Quality Module

Habitat quality is an important component in the study of ecosystem services. The Habitat Quality Model in the InVEST model combines information about land cover and biodiversity threats to generate a Habitat Quality Map [47]. The habitat quality is calculated as follows:

$$Q_{xj}=H_{j}\left[1-(\frac{D_{xj}^{z}}{{K^{z}+D}_{xj}^{z}})\right]$$ (1)

Where: *Q*_*xj*_ is the habitat quality of the grid x in the land use type j, *H*_*j*_ is the habitat adaptability of the land use type j, K is the half-saturated parameter, $D_{xj}^{z}$ is the total threat level of the j habitat grid x, z. The normalization constant is usually 2.5. *D*_*xj*_ is the stress level of grid x in land use type j. The calculation formula is:

$$D_{xj}=\sum_{r=1}^{R}\sum_{y=1}^{Y_{r}}\left(W_{r}/\sum_{r=1}^{R}W_{r}\right)r_{y}i_{yrx}\beta_{x}S_{jr},$$ (2)

Where: R is the stress factor; y is the number of grids in the grid layer of stress factor r; *Y*_*r*_ is the number of grids occupied by stressors; *W*_*r*_ is the weight of stress factor. *r*_*y*_ is the stress factor value of grid y; *β*_*x*_ is the accessibility level of grid x; *S*_*jr*_ is the sensitivity of habitat type j to stress factor r. *i*_*yrx*_ is the stress factor value of grid *r*_*y*_ to the stress level of habitat grid x. This paper sets the weight of stress factors and the maximum impact distance of threat sources by referring to InVEST Model Application Manual 3.2.0 and related studies [48–50] (Table 2). The weight of stress factors is determined by consulting experts. The maximum impact distance and spatial decline type can be defined according to each land-use type in the manual. To determine the specific format of data and the sensitivity index of various land-use types to threat factors (Table 3). The sensitivity index is mainly set according to the actual land use status in the study area.

**Table 2:** Threat sources.

Threat Sources	Influence /km	Weights	Types of Spatial Decay
**Arable Land**	0.5	0.7	linear
**Construction Land**	1	0.5	exponential
**Way**	2	0.6	linear
**Rural Settlement**	2	0.7	exponential
**Urban Settlement**	10	1	exponential

**Table 3:** Threat factor sensitivity.

Land Use Type (primary classification)	Habitat Suitability	Arable Land	Construction Land	Road	Rural Settlement	Urban Settlement
**Arable Land**	0.5	0.2	0.6	0.5	0.35	0.5
**Woodland**	1	0.3	0.8	0.7	0.7	0.6
**Grassland**	0.9	0.5	0.4	0.5	0.55	0.3
**Waters**	1	0.1	1	0.7	0.65	0.8
**Construction- Land**	0	0	0	0	0	0
**Unused Land**	0.7	0.1	0.1	0.2	0.2	0.1

The evaluation of the carbon storage module in the InVEST model includes above-ground carbon, underground carbon, litter carbon, and soil carbon storage [51]. The model uses land-use type as a unit for evaluation, and the total carbon calculation formula is as follows:

$$C_{total}=C_{above}+C_{below}+C_{soil}+C_{dead}$$ (3)

In the formula, *C*_*total*_ is the total carbon storage (t/ha); *C*_*above*_ is the carbon storage of the aboveground part of the vegetation (t/ha); *C*_*below*_ is the carbon storage of the underground part (t/ha); *C*_*soil*_ is the soil carbon storage (t/ ha); *C*_*dead*_ is the carbon storage of vegetation litter (t/ha). The necessary input data in the model include land use/land cover raster dataset; each land use/land cover type stores the carbon density data of 4 parts of above ground, underground root system, soil, and dead organic matter. Among them, the carbon density data is sorted out and analyzed by reading related literature and data [52–56], and according to the actual land-use type of the study area, as shown in the following table (Table 4).

**Table 4:** Carbon density data.

Land Use Type (Secondary Classification)	Aboveground biomass carbon density(t/hm2)	Underground biomass carbon density(t/hm2)	Soil carbon density(t/hm2)	Carbon density of dead organic matter(t/hm2)
**Paddy Field**	5.42	1.96	33.46	0
**Dry Land**	4.7	0	33.46	0
**Woodland**	31.92	3.38	146.82	2.96
**Shrubland**	7.14	3.09	64.29	2
**Sparse woodland**	1.31	2.42	29.9	0.35
**Other woodland**	35.03	7.01	142.58	3.75
**High coverage grassland**	2.33	7.3	43.72	3.08
**Medium coverage grassland**	3.37	7.48	44.36	4.47
**Low coverage grassland**	1.66	3.41	10.93	2
**Lake**	2.75	0	144.13	0
**Beach**	2.3	0	146.26	0
**Town**	0	0	0	0
**Rural settlement**	0	0	0	0
**Construction land**	0	0	0	0

(2) Water Conservation Module

The water conservation module in the InVEST model evaluates the ability of different regions to provide water resources for humans based on the principle of the water cycle and further analyzes the impact of land-use changes on the surface water conservation capacity. The calculation formula is as follows:

$$Y_{x}=\left(1-\frac{{AET}_{x}}{P_{x}}\right)P_{x}$$ (4)

Among them, *AET*_*x*_ is the actual annual average evapotranspiration of grid x, and *P*_*x*_ is the annual rainfall of grid x. For land-use types covered by vegetation, the proportion of evapotranspiration in the water balance is $\frac{{AET}_{\left(x\right)}}{P_{\left(x\right)}}$, and the calculation formula is as follows [57,58]:

$$\frac{{AET}_{\left(x\right)}}{P_{\left(x\right)}}=1+\frac{{PET}_{\left(x\right)}}{P_{x}}-{\left[1+{\left(\frac{{PET}_{\left(x\right)}}{P_{x}}\right)}^{\omega }\right]}^{1/\omega }$$ (5)

$${PET}_{\left(x\right)}=K_{c}(l_{x})\times {ET}_{o}(x)$$ (6)

$$\omega_{\left(x\right)}=Z\frac{{AWC}_{\left(x\right)}}{P_{\left(x\right)}}+1.25$$ (7)

In the formula, *PET*_(*x*)_ is the potential evapotranspiration of grid x, *ω*_(*x*)_ is a non-physical parameter that characterizes the soil characteristics, and is used to correct the annual available water volume of vegetation and the expected precipitation ratio. *ET*_*o*_(*x*) is the reference evapotranspiration of grid x, *K*_*c*_(*l*_*x*_) is the corresponding vegetation evapotranspiration coefficient of vegetation type (*l*_*x*_), and *AWC*_(*x*)_ is the effective water content of vegetation, which is determined by the soil texture Determined by the effective root depth, Z is a Changshu characterizing seasonal factors, and the data fluctuates between one and thirty. For land-use types in areas without vegetation coverage, the actual evapotranspiration calculation formula is as follows:

$${AET}_{\left(x\right)}=Min\left(K_{c}(l_{x})\times {ET}_{o}(x),P_{\left(x\right)}\right)$$ (8)

In the formula, *AET*_(*x*)_ is the actual evapotranspiration of grid x, and *K*_*c*_(*l*_*x*_) is the evapotranspiration coefficient of the land type.

According to previous research results and InVEST model database data, the biophysical parameter Table 5 is obtained [59–62]. The evapotranspiration coefficient is calculated from the above formula.

**Table 5:** Biophysical parameter table.

Land Use Type (primary classification)	Evapotranspiration Coefficient		Root Depth	Actual Evapotranspiration Code
**Arable Land**		1	3000	1
**Woodland**		0.85	6000	1
**Grassland**		0.65	1700	1
**Waters**		1	1000	0
**Construction Land**		0.3	450	0
**Unused Land**		0.2	9	0

(3) Soil conservation module

The soil conservation module in the InVEST model is calculated based on USLE. The necessary parameters are rainfall erosivity factor R, soil erodibility factor K, land use and cover data, vegetation cover and crop management factor C, water and soil conservation measures factor P, and DEM data.

(a) Rainfall erosivity R

Rainfall erosivity R reflects the potential ability of rainfall to cause soil erosion and is an important factor for soil erosion prediction [63]. The calculation formula is as follows:

$$R=\sum_{i=1}^{12}1.735\times 10^{(1.5lg\frac{p_{i}^{2}}{p})-0.8188}$$ (9)

In the formula, P represents the average annual precipitation (mm); *p*_*i*_ is the monthly rainfall (mm). The unit of R in the formula needs to be multiplied by a factor of 17.02 to convert to international common unit.

(b) Soil erodibility factor K

Soil erodibility K is the sensitivity of soil to the erosion and transportation of erosive media such as raindrop splash or surface runoff, and it is a comprehensive manifestation of soil erosion resistance. It is closely related to the combined effects of rainfall, runoff, and infiltration [63,64], Using the soil erodibility factor K value calculation formula developed by Williams in the erosion-productivity impact assessment model (EPIC):

$$K=\{0.2+0.3\times exp[-0.256SAN\times (1-SIL/100)]\}{\left(\frac{SIL}{CLA+SIL}\right)}^{0.3}\times \left(1-0.25\times \frac{C}{C+exp(3.72-2.95C)}\right)\times \left[1-0.7\times \frac{{SN}_{1}}{{SN}_{1}+exp(22.9{SN}_{1}-5.51)}\right]$$ (10)

$${SN}_{1}=1-\frac{SAN}{100}$$ (11)

In the formula, K represents soil erodibility (t·hm2·h·hm-2·MJ-1·mm-1); SAN represents sand content value (%); SIL represents powder content value Size (%); CLA means clay content value (%); C means organic carbon content (%).

(c) Factor C and Factor P

The value of vegetation cover and crop management factor C and soil and water conservation measures factor P is a floating value between 0–1, which is determined by referring to relevant documents and model references [65–67]. As shown in the following Table 6.

**Table 6:** C and P values of different land-use types.

Lucode		Land type	Management Factor C	Support Practice Factor P
**1**	Arable Land		0.23	1
**2**	Woodland		0.05	1
**3**	Grassland		0.3	1
**4**	Waters		0	0
**5**	Construction Land		0	0
**6**	Unused Land		1	1

(d) Soil Conservation

The model uses the revised universal soil loss equation USLE to estimate the amount of regional soil erosion. The calculation formula is as follows:

USLE=R×K×LS×C×P (12)

RKLS=R×K×L (13)

SD=RKLS−USLE (14)

In the formula, USLE is the actual soil loss, RKLS is the potential soil loss, R is the rainfall and runoff factors, K is the soil erodibility factor, LS is the slope length and slope factor, C is the vegetation coverage factor, and P is the water and soil conservation measure factor. SD is the amount of soil conservation.

### Entropy weight method

Entropy weight method is an objective weighting method. In the specific use process, according to the dispersion degree of the data of each index, the entropy weight of each index is calculated by using information entropy, and then the entropy weight is modified according to each index to obtain a more objective index weight [22].

(1) Matrixing of Original Data:


$$X=\left(\begin{array}{ccc} x_{11} & \cdots & x_{1n}\\ \vdots & \ddots & \vdots \\ x_{m1} & \cdots & x_{mn} \end{array}\right)$$ (15)

Use the extreme value method to normalize the matrix to get the matrix:

$$P={\left(p_{ij}\right)}_{m\times n}$$ (16)

The normalization formula is:

$$p_{ij}=\frac{x_{ij}-min\{x_{ij}\}}{max\{x_{ij}\}-min\{x_{ij}\}};p_{ij}=\frac{max\{x_{ij}\}-x_{ij}}{max\{x_{ij}\}-min\{x_{ij}\}}$$ (17)

(2) Define Entropy

The entropy of the i-th index is defined as:

$$H_{i}=-k\sum_{j=1}^{n}f_{ij}lnf_{ij}$$ (18)

$$f_{ij}=\frac{p_{ij}}{\sum_{i=1}^{m}p_{ij}}$$ (19)

(3) Define Entropy Weight


$$W_{i}=\frac{1-H_{i}}{m-\sum_{i=1}^{m}H_{i}}$$ (20)

In the formula, *H*_*i*_ is the entropy of each index, *f*_*ij*_ is the weight of the index value of the i-th item under the j-th index. *W*_*i*_ is the comprehensive weight of the indicator. 0≤*W*_*i*_≤1, $\sum_{i=1}^{m}W_{i}=1$.

## Results

### Habitat quality assessment

**Fig 3:**
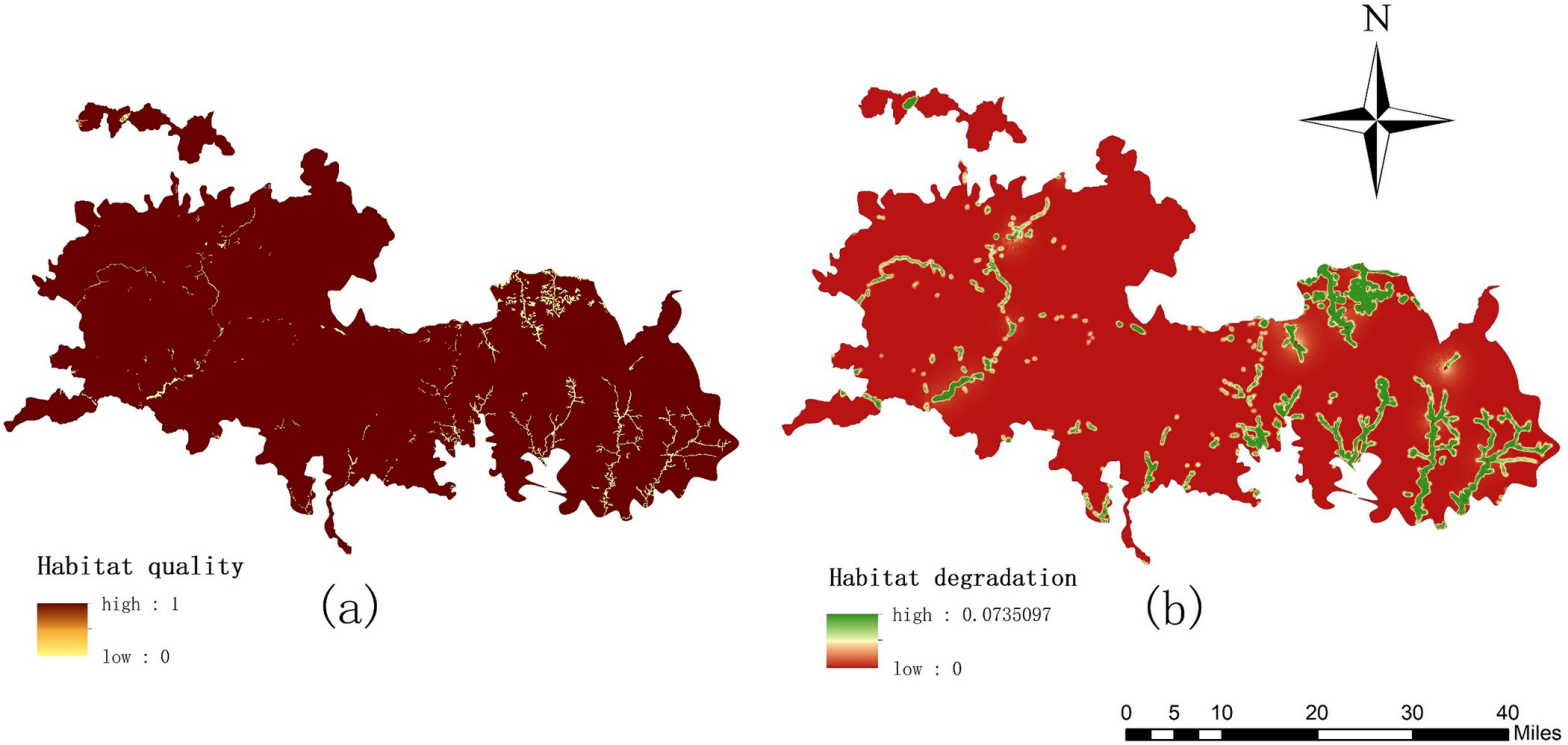
Habitat quality and habitat degradation map.

According to the data required by the model, obtained the spatial distribution map of habitat quality and habitat degradation in Qinling area of giant panda National Park in 2018 (Fig 3). The value range is between 0 and 1. In the habitat quality map, the larger the value, the better the habitat quality, and the smaller the value, the poorer the habitat quality. In the habitat degradation map, the larger the habitat degradation index, the more serious the habitat degradation, the smaller the habitat degradation index, the lower the habitat degradation.

Fig 3(A) shows the spatial distribution map of habitat quality. It can be seen that the overall habitat quality of the study area is relatively high, and the higher-quality areas are concentrated in the middle of the range. These areas are dominated by woodland and grassland, with low human population density, good natural ecological conditions, high vegetation coverage, and high habitat quality. In contrast, the lower areas are distributed on the eastern edge of the study area and are more affected by human beings. Fig 3(B) shows the habitat degradation index map. The habitat degradation index of the study area is generally low. Some areas with high degradation are distributed in the eastern residential area of the study area and are largely affected by human activities.

### Carbon storage assessment

**Fig 4:**
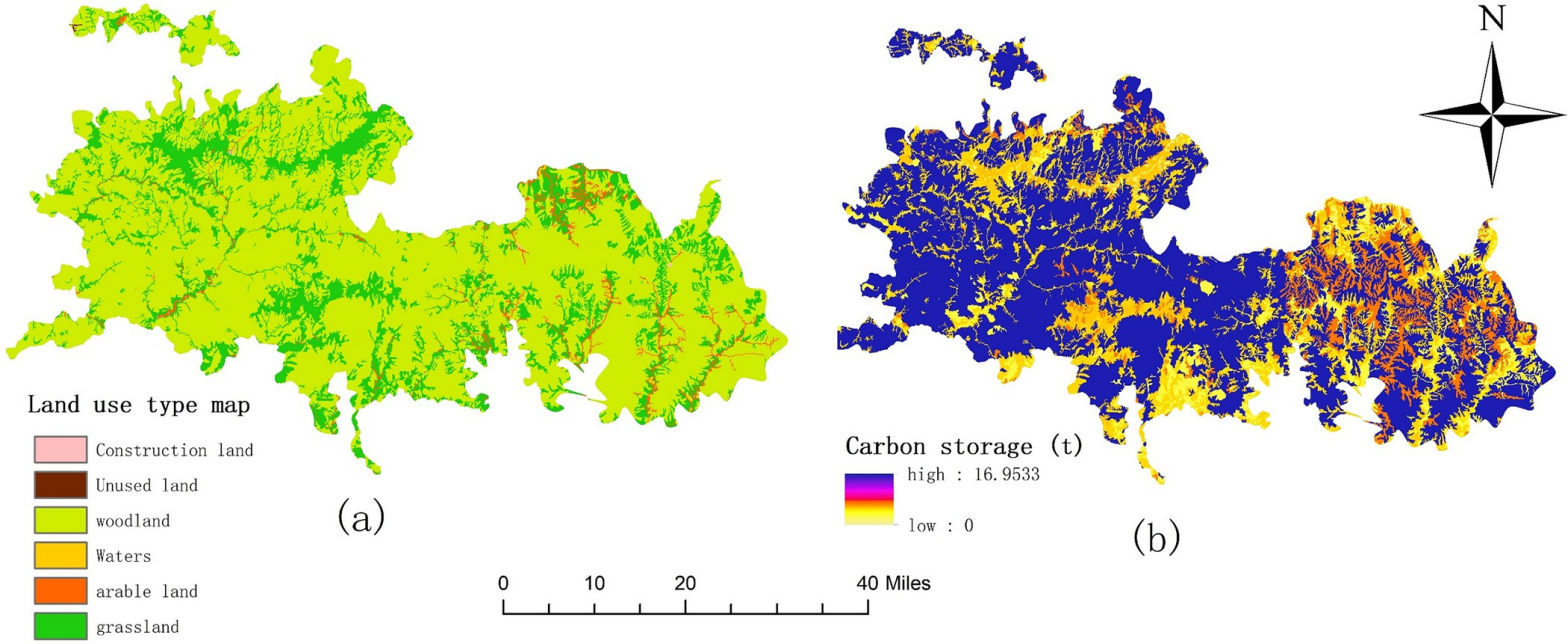
Land use types and spatial distribution of carbon storage.

By running the InVEST model to simulate the carbon storage in the study area, we have mapped the spatial distribution map of carbon reserves in Qinling area of Giant Panda National Park in 2018, as shown in Fig 4. The analysis of the results is shown in the following paragraphs.

According to the operating results of the InVEST carbon storage module, the total carbon storage of the study area in 2018 was 60.5 × 106 t. Because the study area is located in the Qinling Mountains area with high forest coverage and a suitable climate, less interference from human activities, and high carbon storage capacity, the carbon reserve is at a high level. From the perspective of spatial distribution, the carbon storage in the western region of the study area is higher than that in the eastern region. From west to east, the map shows the spatial distribution pattern of high-low-high-low. Since the level of carbon storage is closely related to the distribution of vegetation biomass (forest vegetation carbon storage = vegetation biomass × conversion coefficient), this can reflect the distribution of surface cover to a certain extent. The area of forest land in this region is the most extensive, accounting for 70% of the total area. Combined with the land use type map information, the areas with high carbon reserves are primarily forested areas; the middle value of carbon reserves is mainly distributed in grassland-intensive areas, and the areas with low carbon reserves are mostly cultivated land that is affected by human factors.

### Water conservation assessment

**Fig 5:**
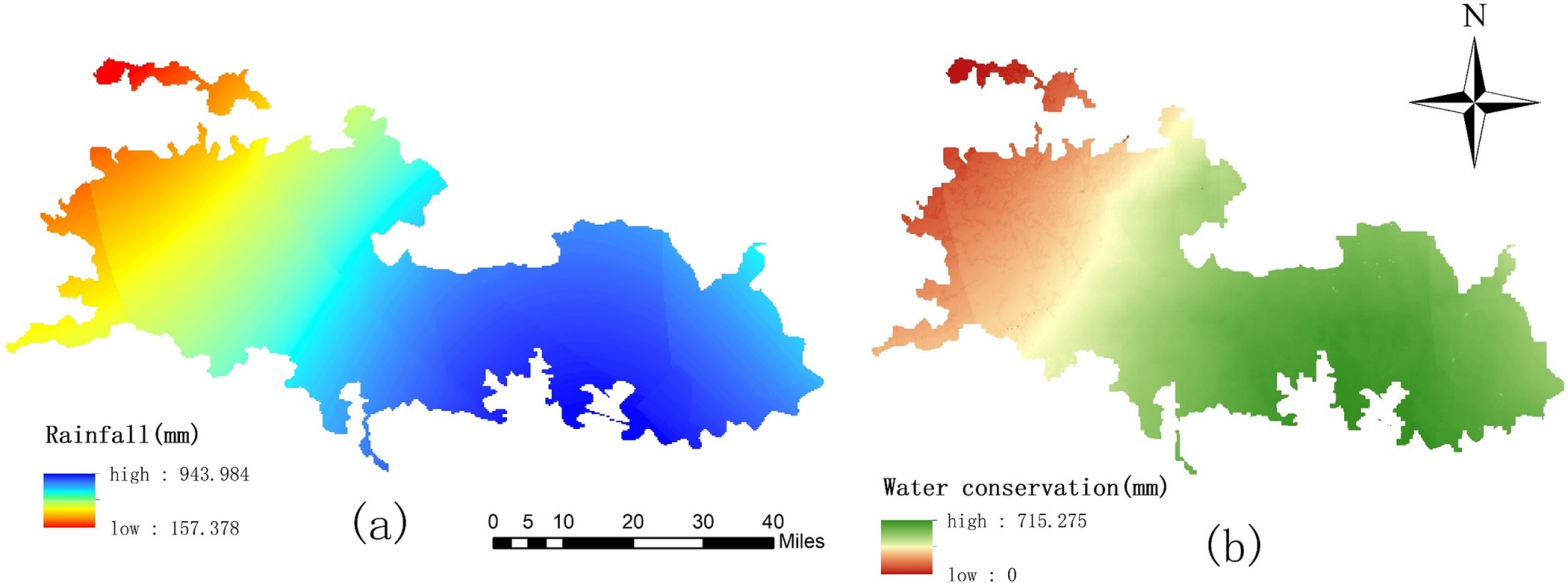
Spatial distribution of annual rainfall and water conservation.

The function of forest water conservation is manifested in many aspects, but mainly in the following aspects: water storage function, runoff regulation function, forest flood reduction, drought resistance function, forest water purification, etc. By running the InVEST model water conservation module, get the spatial distribution map of water conservation in the Qinling area of Giant Panda National Park in 2018, as shown in the Fig 5 below.

### Soil conservation assessment

**Fig 6:**
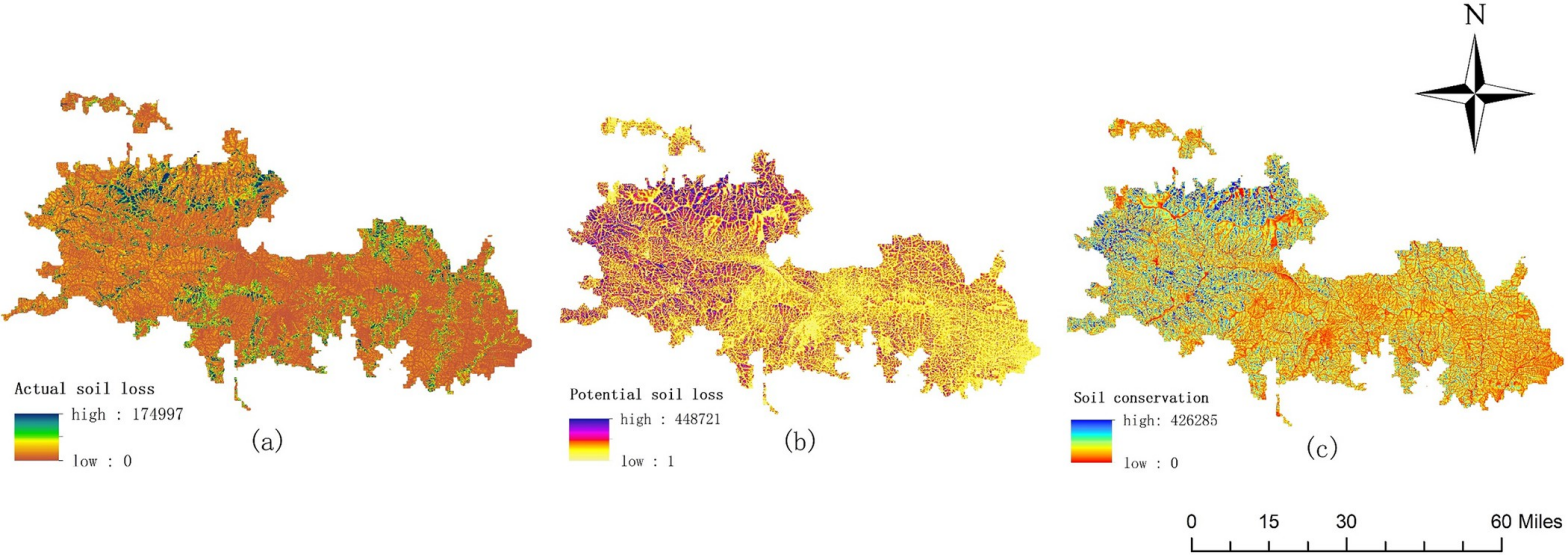
Spatial distribution of soil conservation.

The potential soil erosion, actual soil erosion, and soil conservation in the Qinling area of giant panda National Park in 2018 are obtained by running the invest model, as shown in Fig 6. Potential erosion is the amount of soil erosion simulated by the model in the form of bare land without vegetation coverage, and actual soil erosion is the amount of soil erosion in the state of vegetation coverage and implementation of protection measures.

Fig 6(A) shows the actual soil loss space in the Qinling area of Giant Panda National Park. The total amount of soil loss is 309.3 × 107 t. The actual soil loss in this study area is generally low, being largely distributed in the northern and southern regions of the study area. Fig 6(B) shows the potential soil loss in the Qinling area of Giant Panda National Park, with a total amount of 2866.1 × 107 t. According to the figure, the potential soil loss in the west of the study area is generally higher than that in the east, showing a spatial distribution pattern of high in the west and low in the east. Fig 6(C) shows the amount of soil conservation in the study area. The total amount of soil conservation is 2555.7 × 107 t. In general, the soil conservation in this area showed a decreasing trend from west to east.

### Land-use type and material quality

Since the operation and results of Invest model are based on different land-use types, the four ecosystem services are statistically divided according to different land-use types. The statistical results are as follows in Table 7.

**Table 7:** Statistical results of different land use types.

	habitat quality(index)	carbon storage(t)	water conservation(mm)	soil conservation(t)
**cultivated land**	0.539	434398.97	557.45	412261922.41
**woodland**	0.998	54557266.21	477.41	20611793879.49
**grassland**	0.901	5500834.26	476.65	4510587018.16
**water area**	0.994	4469.72	28.79	2333108.29
**construction land**	0.049	1605.38	442.88	17967637.54
**unused land**	0.706	383.17	3.44	394913.34

Using the zoning statistical function of ArcMap, we calculated the quality of ecosystem services for different land use types in the region (Table 7). The average habitat quality values of different land-use types were as follows: cultivated land:0.539, woodland: 0.998, grassland: 0.901, water area: 0.994, construction land: 0.049, and unused land: 0.706. The carbon storage values of different land use types were as follows: cultivated land: 434398.97 t, woodland: 54557266.21, grassland: 5500834.26 t, water area: 4469.72 t, construction land: 1605.38 t, and unused land: 383.17 t. The water conservation values of different land use types were as follows: cultivated land = 557.45 mm, woodland: 477.41 mm, grassland: 476.65 mm, water area: 28.79 mm, construction land: 442.88 mm, and unused land: 3.44 mm. The soil conservation values of different land use types were as follows: cultivated land: 412261922.41 t, woodland: 20611793879.49 t, grassland: 4510587018.16 t, the water area: 2333108.29 t, construction land: 17967637.54 t, and unused land: 394913.34 t.

### Results of the important assessment of ecosystem services

The entropy weight method was used to calculate the weight of four ecosystem services in Qinling Area of Giant Panda National Park. The weight in descending order was soil conservation: 0.33, carbon storage: 0.26, water conservation: 0.17, and habitat quality: 0.14.

Based on the obtained weight of the ecosystem services function, the importance score is calculated by using the weighted summation formula. The weighted summation method is a statistical analysis method combining quantitative and qualitative evaluation based on the weight to determine the weight of the index and then sum the index score. The calculation formula is as follows [68]:

$$R=\sum_{i=1}^{m}W_{i}S_{i}$$ (21)

Where R is the importance score; M is the number of evaluation indicators; Wi is the weight of each evaluation index; Si is the assignment score of the evaluation index.

**Fig 7:**
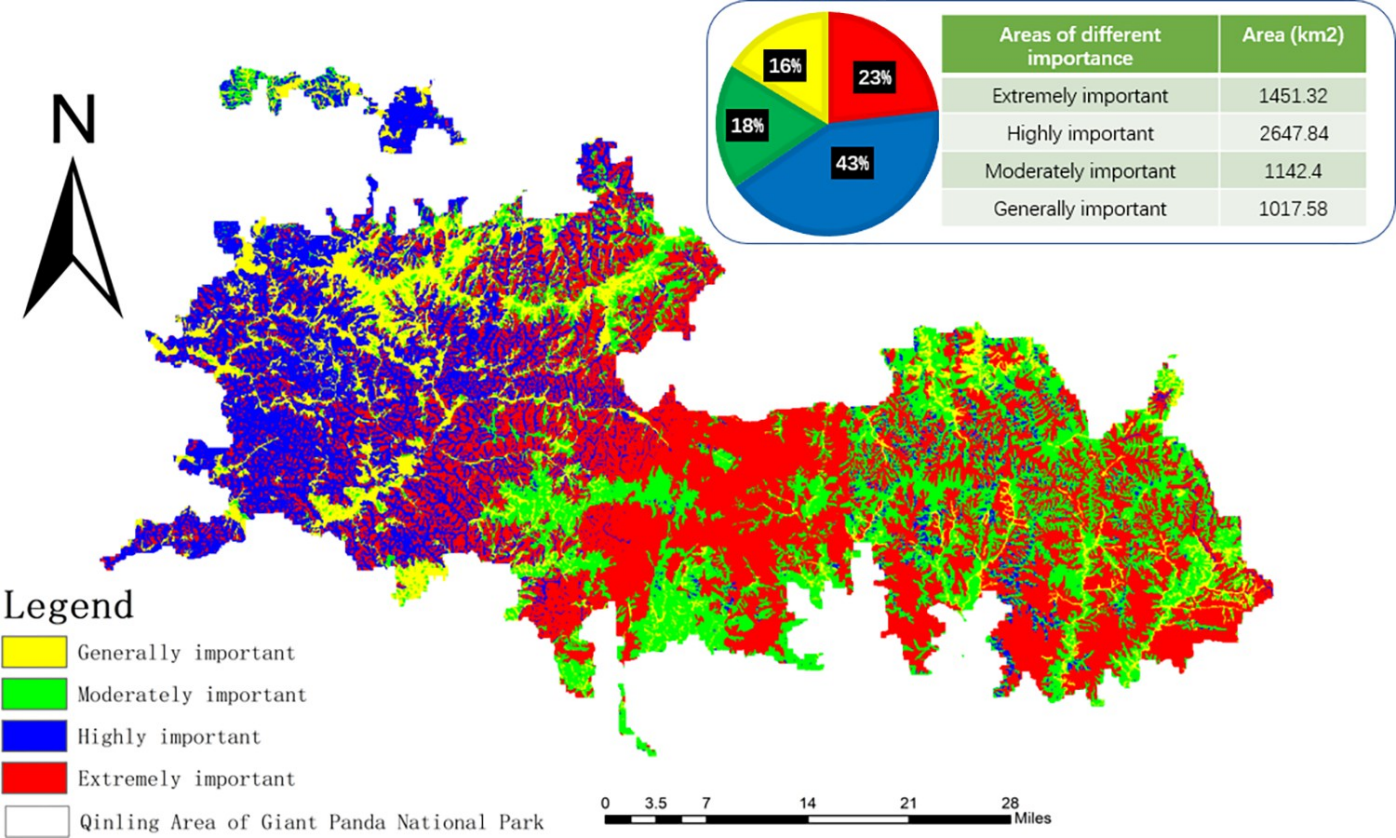
Classification of importance of ecosystem service function.

According to the importance index of The Qinling Area of The Giant Panda National Park, the natural fracture method was used to divide the assessment results into importance grades, and the spatial distribution map of the importance of ecosystem services in the Qinling Area of the Giant Panda National Park was obtained, as shown in Fig 7.

The area of general importance was relatively small at 1017.58 km^2^, accounting for 16% of the research area, and the distribution of this area was wide and scattered. This area includes cultivated and unused land with low carbon storage and habitat quality and simple ecosystem types. When disturbed by nature and other factors, ecological environmental problems in such areas cannot easily be remedied. The area has little impact on the regional ecological environment and features high land utilization, significant development potential, and the lowest ecological protection intensity. The changes in ecological units have little effect on the region’s environmental quality and sustainable development ability, and thus can be ignored. The land use degree of this region is high, and the development potential is excellent.

The moderately important areas were primarily distributed in the eastern parts of the research area, covering an area of 1142.40 km^2^, and accounting for 18% of the total area. The regional ecological system type is unitary, largely including cultivated land and grassland belonging to a fragile ecological environment that is vulnerable to interference by human activities. This area has less influence on the regional ecological environment quality, and little effect on the regulation of the climate. The level of biodiversity is not very high; this should be improved by increasing the regional forest coverage rate, strengthening afforestation, and promoting the development of ecological industry.

The highly important areas were distributed in the west of the study region, with an area of 2647.84 km^2^ accounting for 43% of the total area. These sites are primarily concentrated in the locations with high vegetation coverage in the middle of the study area. If the ecosystem in this region is destroyed, it will affect the quality of the ecological environment and the possibility of sustainable development. However, the degree of impact is slightly lower than that of extremely important area, and the intensity of ecological protection could possibly be slightly lower than that of extremely important area. Vegetation should be protected to prevent soil erosion, and development unrelated to ecological protection should be strictly restricted.

The extremely important areas were distributed throughout the middle of the study site, with an area of 1451.32 km^2^ accounting for 23% of the total area. This portion was dominated by grassland and forest land with high habitat quality, less human interference, and vibrant animal and plant communities with high ecological utility. The region has the functions of water conservation and soil conservation and is an essential habitat for wild animals. Therefore, it is an important territory with respect to providing ecosystem services for human beings. Once this ecosystem unit is destroyed, it will seriously affect the environmental quality of the region. Therefore, it should be regarded as a critical ecological protection region and should continue to implement the policy of afforestation on barren hills, returning farmland to forest, and gradually improving the region’s forest coverage rate to promote the delicate operation of the regional ecosystem.

## Discussion

### Rationality and applicability of the framework

The ecological structure of Giant Panda National Park is complex. To protect and manage the regional ecology, it is necessary to carry out reasonable ecological importance zoning [69]. In the process of ecological importance zoning, there are two key points: one is the selection of ecological zoning indicators, including which indicators to select and which indicators to apply in each level of zoning; the second is the method for determining the boundaries of ecological zoning [70]. Existing ecological zoning studies mostly use expert judgment on these two key points, it can make the partition results show better regularity and are more in line with the goals and requirements of managers [71]. However, this also brings another problem: such a qualitative method is highly subjective, and the choice of experts greatly influences the results [72]. Therefore, it is meaningful to explore how to introduce quantitative methods in these two aspects to reduce the uncertainty caused by qualitative methods for the results of ecological zoning.

In response to this problem, we propose an ecosystem services importance assessment and classification framework, which mainly combines ecosystem services related variables with ecosystem services importance classification. Importantly, we used a known ecosystem service evaluation method (InVEST model) to assess different ecosystem services, divide the study area into high-resolution grid cell data and calculate the weight of each ecosystem service [73,74]. This method of dividing grids has also been applied in ecological risk assessment, land use change and other research [75,76]. While most studies have improved methodologies related to the importance of ecosystem services, However, our research shows that on a large scale, If the classification results of the importance of ecosystem services in a region are subject to greater subjective factors of researchers, it may lead to the reduction of the efficiency of protecting ecosystem services and their benefits [77,78]. Therefore, it is very important to improve the objectivity of the assessment and classification of the importance of ecosystem services in the region.

From the point of view of the purpose of constructing this framework, the framework aims to provide a more objective and scientific method for the division of ecologically important zones and reasonably delineate the ecologically important areas of Giant Panda National Park, combine it with the red line of ecological protection [79]. It improves and supplements the existing ecological zoning system of Giant Panda National Park and provides a reference for further protection and construction of key ecological function protection areas in stages and in batches. From the perspective of the effect of the framework, on the one hand, the framework quantitatively evaluates the selected ecological indicators. On the other hand, the ecological indicators are superimposed to obtain the spatial distribution map of the importance of ecosystem services and the spatial distribution positions of regions of different importance. It is relatively clear that it has a certain value for the refined management of the giant panda national park ecosystem and the differentiated management and control of ecological governance. Thus, the accuracy and objectivity have been greatly improved.

### Enlightenment from the protection and management of Giant Panda National Park

There is abundant research on ecosystem services and their ecological management zoning, which can be summarized as follows: Ecological management zoning is based on different ecological elements, such as water source, wetland, green space, farmland, etc. Ecological management divisions are based on different research scales, such as regions, provinces, typical counties, grid scales, etc. Ecological management zoning is based on different research hotspots, such as ecosystem service cluster zoning, territorial ecological space restoration zoning, ecosystem service supply-demand relationship zoning, etc [80–82]. On the basis of the existing divisions, we divide the research scale into grids, introduce the entropy weight method to determine the weight of ecological indicators, superimpose the four ecological indicators, and divide the important areas. Small-scale ecological sub-regions and second-level and third-level ecological function regions have different regional ecological connotations due to their different regional indicators [83,84]. Therefore, it is necessary to be particularly aware of this point in the use of the ecological zoning system for actual resource management and environmental protection, and understanding the respective ecological connotations is of great significance for the flexible use of the ecological zoning system for effective management [85]. Overall, we have optimized the partitioning method and partitioning scale to make the results of partitioning more scientific.

In this study, the region is divided into four regions with different importance levels, which are the general importance region, medium importance region, high importance region, and extremely important region. The zoning of the importance of ecosystem services makes ecological and environmental protection rise from the previous single biodiversity protection to the protection of the ecological service functions of the entire ecosystem [86]. By evaluating the dominant function of the regional ecosystem, we can distinguish the ecological functional areas that need to be protected and determine the ecological areas that are most worthy of protection with limited resources [87]. Our results show that regions with the same degree of importance are spatially strongly clustered. The purpose of this analysis is to identify the spatial distribution of important areas according to the importance ranking of services. The spatial distribution of the importance of ecosystem services can be used for regional management and planning, and identifying different levels of importance is helpful for targeted protection of the ecological environment in the region.

A general importance area is given priority to unused land and arable land. Water, soil erosion, cultivated land quality improvement, and abandoned land are the main ecological problems left over by a history of land use. Because the total area classified as having general importance cannot avoid artificial interference, it is necessary to set up natural or social buffers, coordinate the relationship between natural and artificial factors, and embark on the protection and improvement of the ecological environment. Therefore, forest vegetation should be restored according to local conditions. The moderately important area is located in the transition area from the general importance area to better habitat, and this portion has a relatively fragile ecological environment that is relatively easy to be disturbed by human activities. This area can be developed with less intensity on the premise of meeting the needs of ecological construction to maintain the natural features as much as possible. This highly important area is a critical ecological conservation location in the Qinling area of Giant Panda National Park. Most of this area is woodland and wetland, and a small portion is arable land. In this area, ecological protection should be considered before the over-all social and economic development—that is, when the social and economic development and ecological protection present a conflict, the requirements of the environ-mental protection should be given priority. This region should maintain ecological diversity that is of great value, and thus we should prioritize water conservation and protection of the vegetation to prevent soil erosion. As a key protected area, the extremely important area has high ecological utility. It plays an essential role in im-proving the species diversity and stability of the ecosystem in the Qinling area of Giant Panda National Park. The site not only has the functions of water conservation and soil conservation but also is a critical wildlife habitat. This area can be used as a reserve to maintain the original ecological environment.

The ecological importance zoning in Giant Panda National Park is based on the analysis of regional ecological characteristics, ecosystem service functions, and the spatial differentiation of ecological importance so as to determine the degree of importance of different ecological areas, which is helpful for decision-makers to target Implement ecological protection and ecological restoration strategies to improve regional ecological security [88].

The service functions of ecosystems are diverse, and the zoning of regional dominant ecological functions cannot completely summarize an ecosystem’s ecological functions. Therefore, it should be combined with the original natural zoning, and the natural reserves and key ecological function areas should be divided based on biodiversity protection [89]. At the same time, we need to strengthen the general survey of resources and ecological environment and formulate a detailed biogeographic flora catalog to improve the existing ecological zoning system.

### Deficiencies and expectations of research

This study has quantified the spatial distribution of ecosystem services in the study area and achieved several significant results. However, because we used only one year’s data, the spatial-temporal changes and future trends of ecosystem services in the study area were not evaluated or predicted. Additionally, the importance assessment and classification of ecosystem services in this study was limited to the four prominent ecosystem services in the study area. Other modules, such as water purification, can be considered in subsequent studies.

## Conclusions

This study constructs an assessment and classification framework for the importance of ecosystem services in Giant Panda National Park, we conduct empirical analysis using land use data, meteorological data, soil data and other data in 2018, and draw the following conclusions:

1. There are obvious differences in the spatial distribution of ecosystem service quality in Giant Panda National Park. The habitat quality index is relatively high and is distributed primarily in the middle of the area. Carbon storage is relatively high, and the region presents a high-low-high-low spatial distribution pattern from west to east. The area with the highest water conservation capacity reached 715.275 mm, increasing in turn from west to east. The soil conservation capacity was 2555.7 × 107 t, showing the spatial distribution pattern of more in the east and less in the west. By analyzing the ecosystem service quality and spatial distribution characteristics of these different ecosystem services, we can better reflect the level and distribution law of the importance of regional ecosystem services protection.
2. The ecosystem service importance zoning and its spatial distribution characteristics of Giant Panda National Park are as follows: The generally important area of the study region is distributed in the northwest, with an area of 1403.94 km^2^. The moderately important area is distributed largely in the east of the study location, with an area of 1041.92 km^2^. The highly important area is distributed in the west of the study location with an area of 2458.68 km^2^. The extremely important area is distributed largely in the middle, with an area of 1199.50 km^2^.
3. The division of ecologically important areas can provide a scientific basis for the classified protection of ecosystem services in giant panda National Park, it also meets the requirements of ecological red line management and control of Giant Panda National Park.

## Supporting information

S1 File (ZIP)
